# Development and function of human cerebral cortex neural networks from pluripotent stem cells *in vitro*

**DOI:** 10.1242/dev.123851

**Published:** 2015-09-15

**Authors:** Peter Kirwan, Benita Turner-Bridger, Manuel Peter, Ayiba Momoh, Devika Arambepola, Hugh P. C. Robinson, Frederick J. Livesey

**Affiliations:** 1 Wellcome Trust/CRUK Gurdon Institute andDepartment of Biochemistry, University of Cambridge, Tennis Court Road, Cambridge CB2 1QN, UK; 2 Department of Physiology, Development and Neuroscience, University of Cambridge, Tennis Court Road, Cambridge CB2 1QN, UK

**Keywords:** Cerebral cortex, Networks, Neural development, Stem cells, Human

## Abstract

A key aspect of nervous system development, including that of the cerebral cortex, is the formation of higher-order neural networks. Developing neural networks undergo several phases with distinct activity patterns *in vivo*, which are thought to prune and fine-tune network connectivity. We report here that human pluripotent stem cell (hPSC)-derived cerebral cortex neurons form large-scale networks that reflect those found in the developing cerebral cortex *in vivo*. Synchronised oscillatory networks develop in a highly stereotyped pattern over several weeks in culture. An initial phase of increasing frequency of oscillations is followed by a phase of decreasing frequency, before giving rise to non-synchronous, ordered activity patterns. hPSC-derived cortical neural networks are excitatory, driven by activation of AMPA- and NMDA-type glutamate receptors, and can undergo NMDA-receptor-mediated plasticity. Investigating single neuron connectivity within PSC-derived cultures, using rabies-based trans-synaptic tracing, we found two broad classes of neuronal connectivity: most neurons have small numbers (<10) of presynaptic inputs, whereas a small set of hub-like neurons have large numbers of synaptic connections (>40). These data demonstrate that the formation of hPSC-derived cortical networks mimics *in vivo* cortical network development and function, demonstrating the utility of *in vitro* systems for mechanistic studies of human forebrain neural network biology.

## Abstract (precis)

**Summary:** Human PSC-derived cerebral cortex neurons form large-scale functional networks that change over time and mimic those found in the developing cerebral cortex *in vivo*.

## INTRODUCTION

The onset of embryonic synapse formation coincides with the development of spontaneous neuronal electrical activity (Katz and Shatz, 1996; Zhang and Poo, 2001). In the developing mouse cerebral cortex, large populations of neurons develop widespread synchronised activity patterns that are oscillatory in their nature (Corlew et al., 2004; Khazipov et al., 2004; Kilb et al., 2011; Leinekugel et al., 2002; Yuste et al., 1992). This synchronised activity results from concentrated bursts of action potentials that lead to large influxes of extracellular calcium (Robinson et al., 1993). Synchronised activity has been observed to develop in a highly specific manner during mouse embryonic development, initiating at ∼E16 and increasing in its frequency, before subsiding by the first week after birth (Corlew et al., 2004). Synchronised bursts in the cortex have been shown to be dependent on glutamatergic synaptic activity (Robinson et al., 1993), indicating that the populations of neurons are forming large-scale excitatory networks. The development of these correlated cortical networks points to a key phase of early network development prior to the onset of sensory experience. The functional role of developmental neuronal oscillations has been an area of extensive research for several decades, with a substantial body of work indicating that oscillations are a mechanism for fine-tuning and strengthening neuronal connectivity (Katz, 1993; Wong, 1993; Wong et al., 1995).

The development of human forebrain synapses and neural networks has not been extensively studied, due to the limitations of acquiring foetal material. Human cortical neurogenesis begins at ∼5 gestational weeks, continuing for ∼100 days, and is mostly completed by 28 weeks (Meyer et al., 2000; Rabinowicz et al., 1996). Synapse formation has been reported to begin between weeks 9 and 10 of human foetal development (de Graaf-Peters and Hadders-Algra, 2006). As is seen in other mammals, neurons undergo a phase of overconnectivity, followed by synaptic pruning and a reduction in neuronal connectivity (Huttenlocher and Dabholkar, 1997; Huttenlocher et al., 1982; Rakic et al., 1986).

Electroencephalography (EEG) recordings of preterm foetuses and electrophysiological analyses of acute slices of foetal human cortex have found that oscillatory bursting activity develops in the cerebral cortex from 20 gestational weeks on, continuing up to birth, after which it eventually diminishes (Dreyfus-Brisac and Larroche, 1971; Khazipov and Luhmann, 2006; Lamblin et al., 1999; Moore et al., 2011; Tolonen et al., 2007). Synchronised bursting also develops in human mixed neuronal cultures derived from embryoid bodies (Heikkilä et al., 2009). Taken together, these data indicate that the acquisition of synchronous firing is also a key property in the development of human cortical neural networks.

Pluripotent stem cells have been used by our group and others to derive synaptically connected, functional human cortical cell types *in vitro* (Espuny-Camacho et al., 2013; Mariani et al., 2012; Shi et al., 2012c). Human pluripotent stem cell (hPSC)-derived cortical neurons provide a system for studying synapse formation as well as the development and function of cortical circuits in humans (Weick et al., 2011). We report here the detailed characterisation of the functional development of human cortical neural networks, together with structural data on the connectivity of those networks. These data serve to validate hPSC cortical cultures as a representative model of developing human cortical networks, and provide a system to uncover new insights into the nature of connectivity in cortical circuits.

## RESULTS

### hPSCs generate adherent 3D cortical neuronal assemblies *in vitro*

**Fig. 1.**
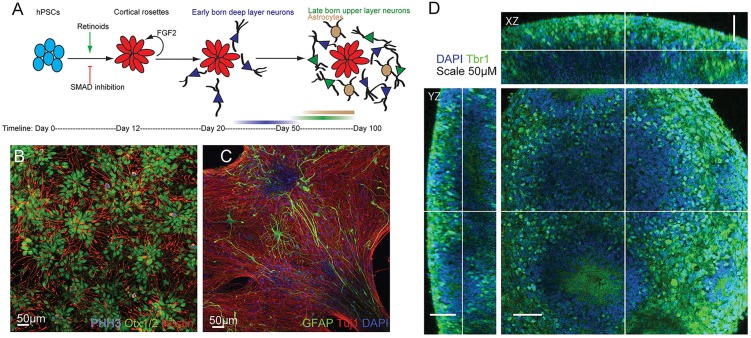
**Generation of cortical neurons and astrocytes from pluripotent stem cells.** (A) Cortical cultures were generated from hPSCs by SMAD inhibition combined with retinoid signalling. (B) Confocal image of day-25 forebrain cortical progenitors expressing OTX1/2, Nestin and phosphohistone H3 (PHH3). (C) Confocal image of day-70 cortical cultures containing GFAP-expressing astrocytes and Tuj1^+^ neurons. (D) Two-photon confocal image of day-72 cortical neurons showing 3D cultures expressing the deep layer cortical transcription factor Tbr1.

In order to study the development of network properties in hPSC neural networks, cortical projection neurons were generated using our previously published method (Fig. 1A) (Shi et al., 2012a,c). We used dual SMAD inhibition in combination with retinoid signalling to generate polarised neuroepithelial rosettes with a dorsal forebrain identity (Fig. 1B). Over several weeks *in vitro*, hPSC-derived forebrain progenitor cells give rise to the major subtypes of deep and upper layer glutamatergic cortical neurons in approximately equal proportions (Shi et al., 2012a,b,c) in the same temporal order that is observed in mammalian cortical development (Molyneaux et al., 2007) (Fig. 1A). Towards the latter end of neurogenesis, astrocytes are generated (Fig. 1C). Two-photon microscopy revealed that our forebrain cultures consist of adherent three-dimensional (3D) neuronal assemblies that range between 10 μm and 200 μm in thickness (Fig. 1D). The presence of diverse populations of cortical neurons and astrocytes in adherent 3D assemblies suggests that hPSC-derived cortical cultures could act as a useful model system for studying human neuronal circuit development *in vitro*.

### Electrical properties of PSC-derived human cortical neurons mature over time

**Fig. 2.**
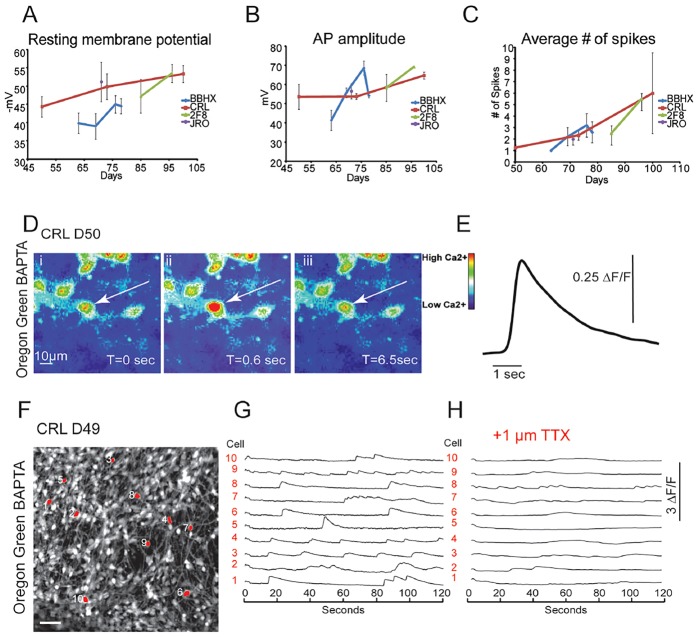
**Electrical maturation of hPSC-derived cortical neurons over weeks *in vitro*.** (A-C) Whole-cell electrophysiological recordings in four hPSC lines (total of *n*=51 cells) showing the maturation of (A) resting membrane potential, (B) action potential amplitude and (C) average number of spikes fired during 1-s 10-pA current injection. (D,E) Use of calcium indicator as a proxy for neuronal firing. (D) Snapshots of a time series of cortical neurons loaded with calcium indicator Oregon Green BAPTA 488 (OGB), demonstrating dynamic changes in intracellular calcium. (E) Trace showing the change in fluorescence (ΔF/F) over time in the neuron highlighted by the arrow in D. (F-H) Action potentials in OGB-loaded cortical neurons were blocked with the sodium channel blocker tetrodotoxin (TTX). Scale bar in F: 50 μm.

To investigate the functional development of cortical neurons *in vitro*, we performed whole-cell electrophysiological recordings over a period of 9 weeks in four iPS cell lines. Analysis of these recordings revealed a progressive maturation of whole-cell and action-potential firing properties over this time period: a reduction in resting membrane potential (Fig. 2A), an increase in action potential amplitude (Fig. 2B), and an increase in the number of action potentials fired per cell in response to 10 pA current stimuli (Fig. 2C; supplementary material Table S1). These data support our previous findings that hPSC-derived cortical neurons acquire mature firing properties *in vitro* (Shi et al., 2012c).

Although whole-cell electrophysiology provides excellent resolution for the study of neuronal properties and synaptic connections at the cellular level, it does not provide the optimal coverage to study the network dynamics of large populations of cells. By contrast, calcium imaging allows for the detection of neuronal activity across a population of cells and at single-cell resolution (Ikegaya et al., 2005). Calcium imaging serves as a reliable proxy for neuronal activity, with the number of action potentials fired in a burst by a neuron directly correlated to the size of the observed calcium transient (Robinson et al., 1993; Smetters et al., 1999). We found that by 7 weeks in culture, hPSC-derived cortical neurons loaded with the calcium indicator dye Oregon Green BAPTA-1 488 (OGB) (Fig. 2D) exhibited calcium transients that showed fast onset (<0.6 s) and slow decay (ranging from ∼1 to 8 s) (Fig. 2E). Transients recorded across ensembles of hPSC-derived cortical neurons (Fig. 2F,G) were blocked in the presence of tetrodotoxin, the voltage-gated sodium channel antagonist (Fig. 2H), indicating that the calcium transients were due to neuronal activity across the population.

### Developmental changes in network activity in hPSC-derived cortical cultures

**Fig. 3.**
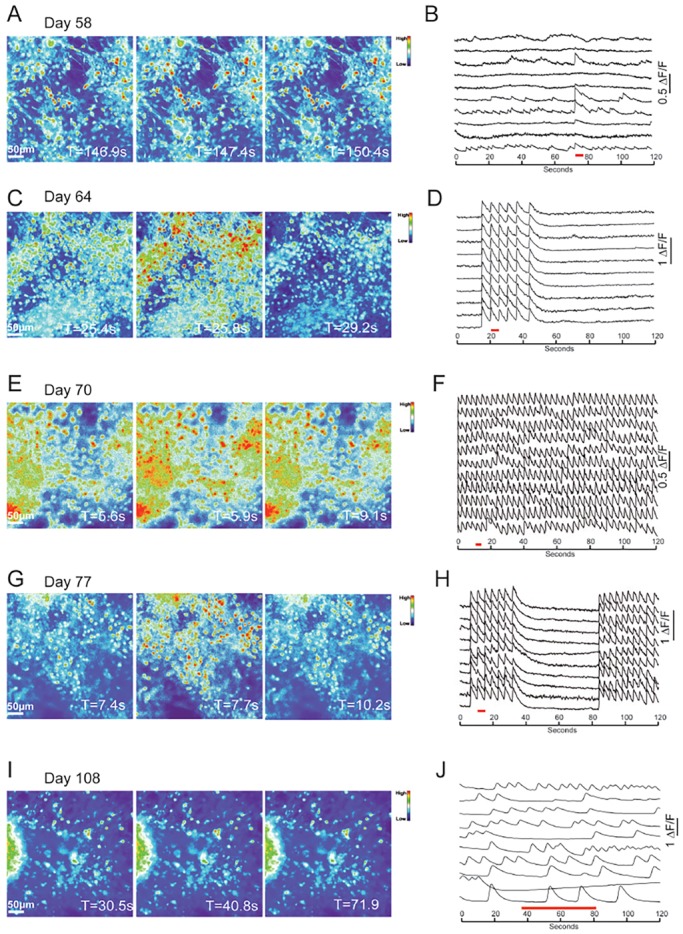
**Synchronised network development in hPSC-derived cortical cultures.** (A,C,E,G) Frames of calcium recordings showing synchronous firing at days 58, 64, 70 and 77, and (I) asynchronous firing on day 108 in cells loaded with OGB. (B) ΔF/F trace demonstrating that small numbers of cortical neurons in day-58 cultures fire synchronised transients. (D) By day 64, the majority of OGB-labelled cells participate in synchronised firing, and transients are more frequent. (F) The frequency of synchronised firing peaks around day 70, and (H) declines by day 77. (J) By day 108, neuronal activity is asynchronous. On each trace, the time period represented by the frames in A,C,E and G is highlighted by a red line.

By week 8 after cortical induction, we observed coherent, synchronised firing among large numbers of cortical neurons (Fig. 3). Synchronised firing has been observed in developing neural networks *in vivo* (Khazipov et al., 2004; Kilb et al., 2011; Leinekugel et al., 2002), in acute *ex vivo* cortical slices (Corlew et al., 2004), as well as in dissociated primary cortical cultures *in vitro* (Corlew et al., 2004; Robinson et al., 1993), evolving in a highly stereotyped manner: first, a phase of uncorrelated neuronal firing, followed by a phase of oscillatory synchronised network firing (Corlew et al., 2004), which is replaced by a phase of complex network activity.

Performing a longitudinal analysis of network firing from day 50, up to >160 days in culture, we found that hPSC-derived cortical cultures undergo the same stereotyped developmental progression. Synchronised firing (bursting, or oscillation) was first observed after 58 days in culture (Fig. 3A,B; supplementary material Movie 1). Synchronicity was rare at this stage, with bursting occurring in less than half of the cells in a given field of view, and at a low frequency (5×10^−4^ Hz). By day 64, almost all of the cells sampled participated in synchronised firing (Fig. 3C,D; supplementary material Movie 2). The bursts had a higher frequency (0.024 Hz), with long periods of inactivity between bursts. By day 70, the level of synchronisation peaked, with few inter-burst intervals and a high frequency of bursting (0.18 Hz; Fig. 3E,F; supplementary material Movie 3), defined as the number of bursting events per unit time. With a further week in culture, synchronicity decreased, with longer time periods between transients, and a reduction in the frequency of correlated bursts (0.17 Hz; Fig. 3G,H; supplementary material Movie 4). The reduction in synchronous firing continued over time, such that bursting was rare past day 100 (0.011 Hz, day 100; 0 Hz, day 108; 0.016 Hz, day 112), replaced by complex patterns of neuronal activity (Fig. 3I,J; supplementary material Movie 5).

The sequence of network behaviours described above was reproduced in three cortical inductions from two hPSC lines, in a total of six cultures per cortical induction, monitored for 158 days in culture. Bursting activity developed in all three inductions and in all dishes from each induction. This revealed variation in the timing at which bursting developed, as well as the peak bursting frequency that was reached. On two occasions bursting occurred between 8 and 16 weeks after induction, while on a third occasion the period of bursting spanned between 14 and 23 weeks in culture. Despite this variation, the same developmental pattern of coherent firing was observed in the three sets of cultures, increasing to a peak frequency over 3-6 weeks and then diminishing towards zero over a further 2-5 week period, to be replaced by complex neuronal firing patterns. Therefore, coherent firing develops in PSC-derived cortical neural networks in the same stereotypical order that occurs in the cerebral cortex *in vivo*, although with some variability in onset (Corlew et al., 2004; Kilb et al., 2011).

### Expression of glutamate receptor subunits and dendritic spine development

**Fig. 4.**
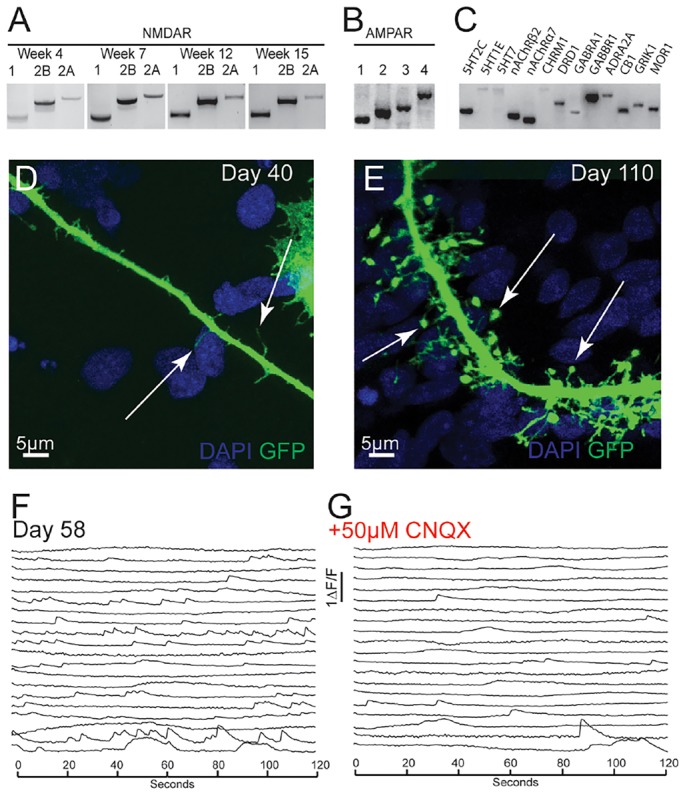
**Functional synaptic development in hPSC-derived cortical cultures (CRL and NDC lines).** (A) RT-PCR for NMDA receptor subunits 1, 2B and 2A at weeks 4, 7, 12 and 15. (B) RT-PCR for AMPA receptor subunits 1, 2, 3 and 4 in week-10 cultures. (C) RT-PCR for cortical neurotransmitter receptors in week-10 hPSC-derived cortical cultures: serotonin receptors 2C, 1E, 7 (5HT2C, 5HT1E, 5HT7); nicotinic acetylcholine receptors β2 and α7 (nAChRβ2, nAChRα7); muscarinic acetylcholine receptor 1 (CHRM1); dopamine receptor 1 (DRD1); GABA-A receptor subunit α1 (GABRA1); GABA-B receptor subunit 1 (GABBR1); adrenoreceptor α2A (ADRA2A); cannabinoid receptor 1 (CB1); glutamate receptor ionotrophic kainate 1 (GRIK1); opioid receptor μ1 (MOR1). (D) Immature filopodial dendritic spines (arrows) in day-40 cortical neurons infected with GFP-expressing lentivirus. (E) Mature dendritic spines (arrows) in day-110 cortical neurons infected with GFP-expressing lentivirus. (F) Calcium recordings on day-58 cultures. (G) The majority of neuronal activity is blocked by AMPA receptor antagonist CNQX.

In the cerebral cortex, projection neuron neurotransmission is glutamatergic and excitatory, with interneuron-mediated, GABAergic inhibition playing an important modulatory role. A number of other neurotransmitters are important for cortical function, including serotonin, acetylcholine and dopamine (McCormick, 1992). RT-PCR was used to determine which glutamate neurotransmitter receptors were expressed in stem cell-derived cortical systems. This showed that many of the major subunits of the NMDA and AMPA receptors were expressed (Fig. 4A,B). Notably, the NR2A NMDA receptor subunit that is mostly expressed in the adult brain (Sheng et al., 1994) was detected from as early as week 4, and was still present in mature week-15 cultures (Fig. 4A). In addition, the foetally expressed NR2B subunit was also present. These data indicate that hPSC-derived cortical cultures contained a mixture of mature and immature NMDA receptor subunits, potentially reflecting the mixture of neurons of different developmental ages present in this system (Shi et al., 2012c).

In addition to the major excitatory receptor subunits, we found that subunits of GABA, acetylcholine, dopamine, serotonin, cannabinoid, adrenergic and opioid receptors, were also expressed by human cortical neurons at this stage (Fig. 4C). The expression of neurotransmitter receptor subunits in hPSC-derived cortical cultures indicates that neurotransmitter receptors required for normal cortical synaptic function are present.

Dendritic spines are important post-synaptic scaffolds for receiving excitatory synaptic inputs and are a hallmark of functionally mature neurons (Yuste, 2011). To investigate the development of dendritic spines in hPSC-derived cortical networks, individual neurons were labelled by infecting them with a GFP-expressing lentivirus. Young cortical neurons (day 40) contained many immature filopodial spines, thin protrusions from the dendrite that were ∼2-5 μm in length (Fig. 4D). By day 110, the dendrites had many morphologically mature spines, with thin necks (∼1-6 μm in length) and round heads (Fig. 4E). These data indicate that hPSC-derived cortical neurons develop the appropriate physical scaffolds for excitatory synapses in a manner similar to what is seen *in vivo* (Calabrese et al., 2006), by first forming thin filopodial spines and then developing into morphologically mature spines.

We have previously reported that human forebrain cultures develop functional synaptic properties from ∼50 days after neural induction (Shi et al., 2012c). We observed a reduction in the number of calcium transients in the presence of AMPA receptor antagonist CNQX in 58-day-old cultures (Fig. 4F,G). As expected, action potentials were still present when synaptic activity was blocked (Fig. 4F), confirming that the neurons could still generate spontaneous action potentials in the absence of functional synapses. These data confirm that functional synapses are present at this stage, and that a large proportion of the firing activity that we observe in populations of cortical neurons by calcium imaging was a result of excitatory network activity.

### Pharmacology of hPSC-derived cortical networks

**Fig. 5.**
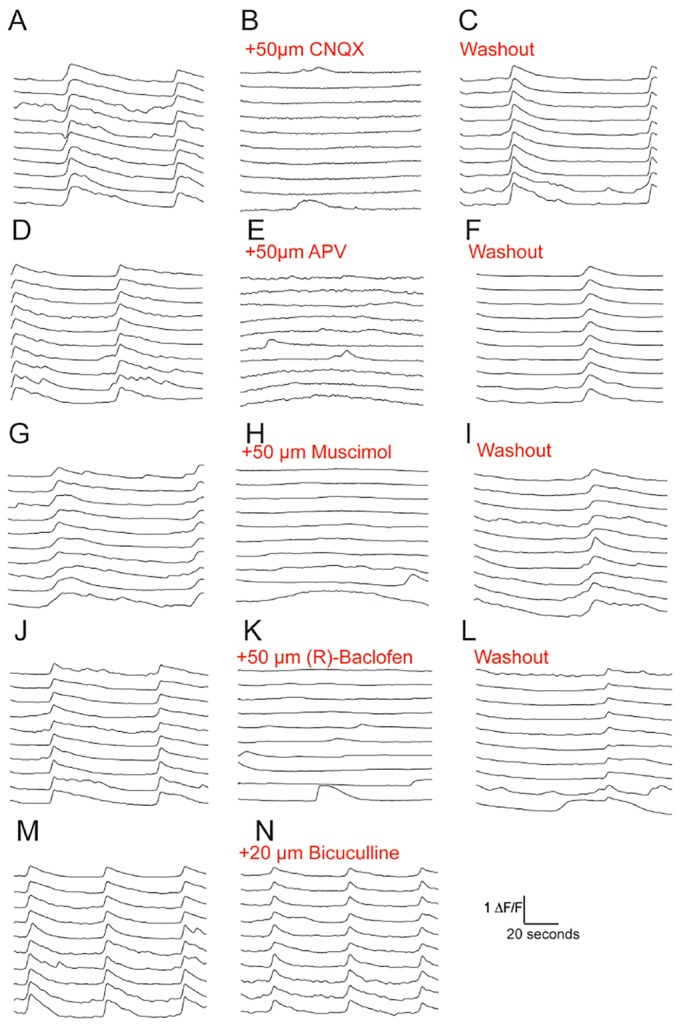
**Pharmacology of hPSC-derived cortical networks.** (A-C) Synchronised firing is blocked by the AMPA receptor antagonist CNQX and returns after washout of CNQX. (D-F) NMDA receptor antagonist APV blocks all synchronous activity. Synchronised transients return after washout of APV. (G-I) In the presence of the GABA-A receptor agonist muscimol, synchronised transients were blocked, but returned after washout. (J-L) GABA-B receptor agonist R-baclofen blocked synchronised activity. Synchronous transients returned after washout. (M,N) There was no change in synchronised firing in the presence of GABA-A receptor antagonist bicuculline.

Synchronised activity *in vivo* and *in vitro* has been previously shown to be due to excitatory, synaptically driven network activity (Corlew et al., 2004; Robinson et al., 1993). We found that all synchronous activity was blocked in the presence of the AMPA and NMDA receptor antagonists CNQX and APV (Fig. 5A-F). As expected, asynchronous calcium transients were still present in cultures treated with CNQX and APV (Fig. 5B,E). These data demonstrate that the synchronous calcium transients that we observe in hPSC-derived cortical cultures are a result of excitatory network activity.

Inhibition mediated by GABAergic interneurons plays a pivotal role in the control of excitatory neuron activity and networks (Isaacson and Scanziani, 2011). Agonist-mediated activation of GABA-A (muscimol) and GABA-B (R-baclofen) receptors led to a complete suppression of synchronised firing in hPSC-derived cortical networks (Fig. 5G-L). This showed that cortical networks respond appropriately for mature neurons to GABA-A and GABA-B receptor activation by inhibiting neuronal activity. We have previously observed that GABAergic interneurons are present in very small numbers in hPSC-derived cortical cultures (Shi et al., 2012c). Primary mouse cultures containing both excitatory and inhibitory neurons have been shown to increase transient sizes as well as interburst intervals when GABAergic activity is blocked (Robinson et al., 1993). We observed no change in synchronised activity in hPSC-derived cortical cultures in response to GABA-A receptor antagonist bicuculline (Fig. 5M,N). This indicates that the hPSC-derived cortical networks that we observed were exclusively excitatory, with no detectable influence of inhibition on network properties.

### Induction of NMDA receptor-dependent long-term potentiation in human stem cell-derived neural networks

**Fig. 6.**
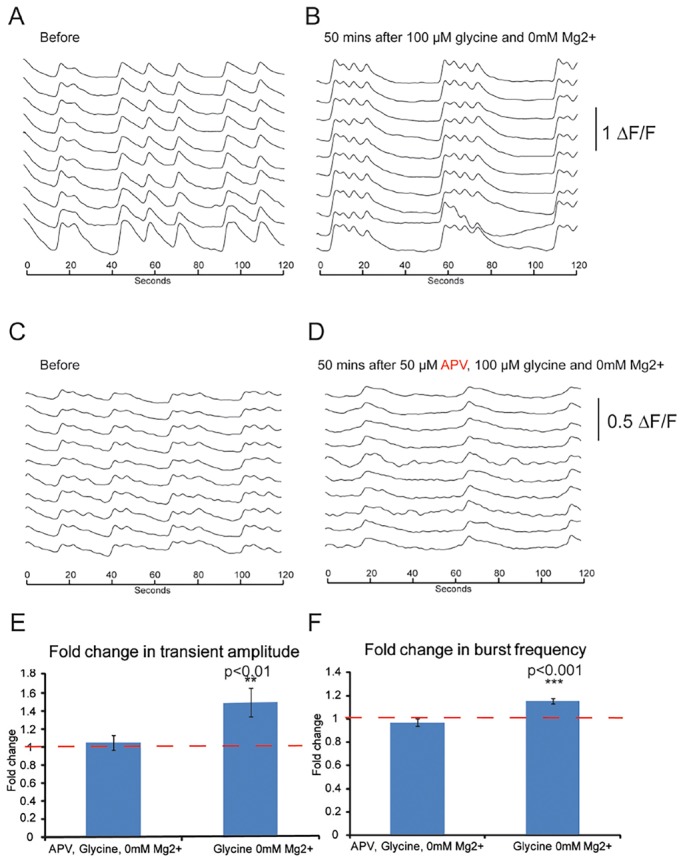
**Plasticity in iPSC-derived cortical neural networks.** (A) Synchronised activity in day-145 CRL iPSC-derived cortical cultures. (B) 50 min after a 5-min treatment with 100 µM glycine and 0 mM Mg^2+^, cultures exhibited a marked change in their pattern of synchronised bursting. (C,D) Synchronised activity before (C) and 50 min after (D) treatment with APV during a 5-min pulse with 100 µM glycine and 0 mM Mg^2+^ solution. (E) The amplitude of synchronised transients showed a significant increase (*P*<0.01, Student's *t*-test) after glycine and 0 mM Mg^2+^ treatment, and no significant change with APV/glycine/0 mM Mg^2+^ treatment. (F) Significant increase (*P*<0.01, Student's *t*-test) in the frequency of bursts after glycine/0 mM Mg^2+^ treatment, and no significant change after APV/glycine/0 mM Mg^2+^ treatment.

To analyse the functional maturity of stem cell-derived neural networks further, we investigated whether these neurons and networks could support NMDA-receptor mediated plasticity. To do so, we used a standard approach to increase NMDA-receptor activation, referred to as chemical long-term potentiation, during the periods of synchronised neuronal activity (Lu et al., 2001) (Fig. 6A,B).

This stimulation protocol led to increases in both the frequency and amplitude of neuronal calcium transients (Fig. 6E,F), and both of these increases were blocked by co-administration of an NMDA receptor antagonist (APV; Fig. 6C-F). In addition to demonstrating that stem cell-derived neural networks can undergo synaptic plasticity, these experiments also confirm that the NMDA receptors expressed by stem cell-derived cortical neurons are functional.

### Connectivity in hPSC-derived cortical networks

Non-random patterns of neural connectivity are a feature of the cerebral cortex and across the nervous system (Brown and Hestrin, 2009; Song et al., 2005). In the cortex, ordered microcircuits exist that are composed of specific connections between particular subclasses of neurons (Yoshimura and Callaway, 2005; Yoshimura et al., 2005), as well as between neurons that are clonally related (Yu et al., 2009, 2012). Computational modelling of connectivity in the cortex suggests that networks adhere to a scale-free topology, which includes small numbers of hyper-connected hub-like nodes, and a large number of neurons with few connections (Eguíluz et al., 2005; Feldt et al., 2011). In particular, scale-free connectivity appears to be an important feature of synchronised networks (Batista et al., 2007; Grinstein and Linsker, 2005).

**Fig. 7.**
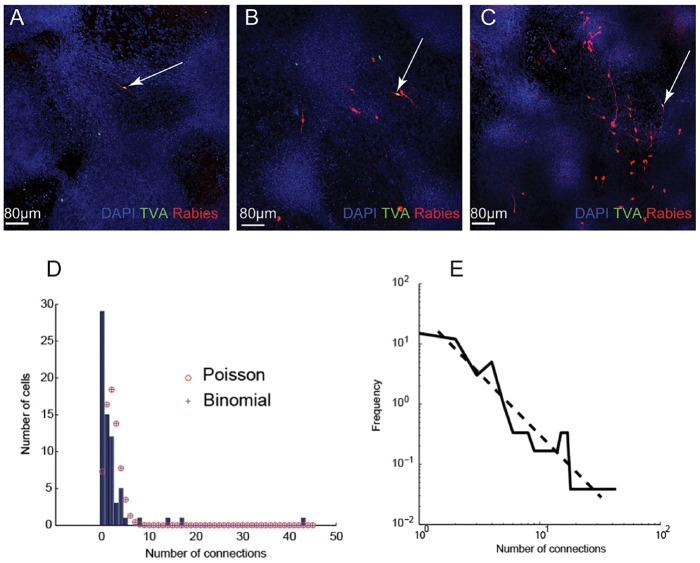
**Analysis of single neuron connectivity in stem cell-derived cortical networks.** (A-C) Examples of neurons infected with pseudotyped rabies virus, showing (A) no connectivity; (B) intermediate levels of connectivity; (C) high levels of connectivity. The primary postsynaptic neurons (arrows) are positive for TVA-GFP-expressing and RFP-expressing rabies virus, whereas the presynaptic cells only express the RFP rabies virus. (D) Histogram of the frequency of different numbers of connections, showing that the distribution of connectivity does not fit a Poisson or binomial distribution. The Poisson distribution with the same mean number of connections (λ=2.25 connections per neuron) is shown in red. The best fit binomial distribution (*n=*735, *P=*0.0031) is shown as blue crosses. (E) The data fit with a scale-free distribution, with a power of −2.

To determine the patterns of connectivity in hPSC-derived cortical networks, we performed single-neuron trans-synaptic tracing in synchronised cultures at day 70 (Luo et al., 2008; Wickersham et al., 2007). We observed a range of connectivity levels, from 0 connections to 43 presynaptic inputs per neuron (Fig. 7A-C). Of the 69 neurons sampled, the mean number of presynaptic connections was low, 2.25 connections per neurons. The vast majority of neurons (*n*=66) had between 0 and 8 connections, whereas three had 14, 17 and 43 connections, respectively (Fig. 7D).

We found that this dataset did not fit a binomial or Poisson distribution (Fig. 7D), but instead followed a scale-free distribution (Fig. 7E). This distribution suggested that hPSC-derived cortical networks consist of large numbers of neurons with few connections, and a small number of highly connected cells that act as hubs. Allowing for the underrepresentation of connectivity using pseudotyped rabies monosynaptic tracing (Callaway and Luo, 2015), these data suggest that *in vitro* human cortical networks have similar connectivity patterns to cortical neurons *in vivo*.

### Calcium imaging of neuronal firing identifies structured network activity at late stages of development

**Fig. 8.**
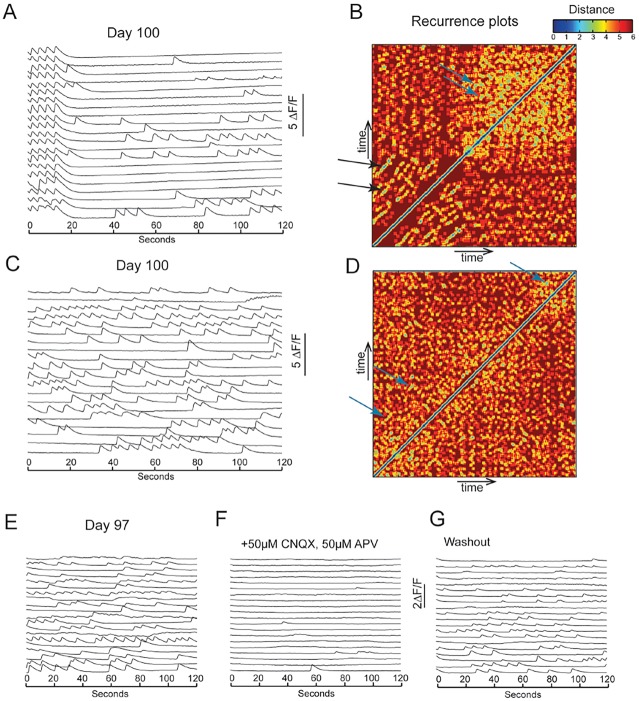
**Structured network activity in late-stage hPSC-derived cortical cultures.** (A) ΔF/F trace of a calcium recording of day 100 cortical cultures, showing synchronised bursts followed by dynamic spontaneous firing patterns. (B) Recurrence plot of the recording shown in A. Recurrence plots are temporal heatmaps of firing sequences between two given time points. Firing sequences that are recurring are shown as blue. Firing sequences with low recurrence are shown as red. A high level of recurrence was evident during synchrony (black arrows). Non-synchronised dynamic firing also showed recurrence (blue arrows). (C) ΔF/F trace of a calcium recording on day-100 cultures, no longer showing synchronisation. (D) Recurrence plot of the recording shown in C. Neuronal firing patterns had high levels of recurrence (blue arrows). (E-G) Complex firing patterns were blocked with AMPAR and NMDAR antagonists CNQX and APV. The complex firing returned after washout of the drugs.

During development, large-scale cortical circuits develop into sparsely connected and highly specific circuits (Song et al., 2005; Thomson et al., 2002; Yoshimura and Callaway, 2005; Yoshimura et al., 2005). In late-stage cultures (week 16) we observed highly dynamic and complex firing patterns (Fig. 8; supplementary material Movie 5). To investigate the nature of the complex firing patterns, we performed recurrence analyses to test whether repeating patterns of activity could be observed in the cultures. This analysis showed that synchronised firing had high levels of recurrence (Fig. 8A,B). During asynchronous complex firing, there was also a high level of recurrence (Fig. 8C,D), indicating that activity at this mature stage was highly structured. In one long, stable complex time series (day 100), we used the technique of random surrogates (see Materials and Methods) to show that the recurrence of this complex activity was highly significant (*P*<0.001).

To demonstrate that complex firing was dependent on functional excitatory synapses between neurons, the AMPA and NMDA receptor antagonists CNQX and APV were added to mature cultures that exhibited complex firing patterns. Under these conditions, complex network activity was blocked (Fig. 8E,G), with ordered firing returning upon washout, indicating that glutamatergic transmission was essential for complex activity (Fig. 8F). Together, these results suggest that, after the decline of synchronised activity, highly structured excitatory networks begin to emerge in this system, suggestive of the development of ordered connectivity.

## DISCUSSION

We report here the characterisation of cerebral cortex neural network development and function from human pluripotent stem cells. We have previously shown that individual cortical neurons acquire mature firing properties over several weeks *in vitro* (Shi et al., 2012c). We now find that at relatively early stages of cortical development, when the overwhelming majority of neurons are layer 5 and layer 6 corticospinal and corticothalamic neurons, functional glutamatergic, synaptically connected neural networks are present. The functional properties of those networks change over time, in a manner that reflects network development *in vivo*. We therefore conclude that directed differentiation of pluripotent stem cells to cerebral cortex neurons recapitulates major features of neural network development, in addition to replaying accurately the temporal order of neurogenesis.

Rodent and human cerebral cortex neural networks undergo several distinct phases of activity during foetal development. Oscillations, or bursting activity, appear in the developing human cerebral cortex from approximately halfway through gestation and continue until birth, disappearing in the early postnatal period (Dreyfus-Brisac and Larroche, 1971; Khazipov and Luhmann, 2006; Lamblin et al., 1999; Moore et al., 2011; Tolonen et al., 2007). In this *in vitro* system, oscillations appear ∼1 month after the onset of neurogenesis, which overlaps with layer 2-4 neurogenesis in this system (Shi et al., 2012c). The equivalent human gestational age is approximately week 15-18 (Finlay and Darlington, 1995). Oscillations depend on glutamatergic transmission, as they were completely halted by glutamate receptor antagonism.

Although oscillations have been reported in mixed human neuronal cultures (Heikkilä et al., 2009), the orderly development of network activity in this *in vitro* excitatory cortical system is striking, given the lack of cortical lamination and the absence of inhibitory interneurons. The effect of the lack of organisation of projection neurons into discrete cortical laminae in this system on the specificity of connectivity is currently unknown, although it is noteworthy that the *Reeler* mouse cortex, in which cortical lamination is markedly disrupted, appears to function broadly normally (Simmons et al., 1982; Simmons and Pearlman, 1982, 1983). Therefore, it is possible that the formation of functional cortical networks does not depend on orderly lamination and might depend on specific cell recognition cues to promote the specificity of synaptic connectivity, as has been shown *in vitro* for primary cultures of mouse hippocampal neurons (Williams et al., 2011).

Current theory suggests that inhibition is required for the emergence of oscillations (Buzsaki, 2006). The system used here generates almost exclusively excitatory, glutamatergic projection neurons (Shi et al., 2012c) and thus lacks inhibitory input. There are functional GABA receptors expressed by the projection neurons, as GABA agonists completely suppress neuronal activity, including oscillations. However, GABA antagonists have no effect on neuronal firing or oscillations, demonstrating that GABA does not contribute to network oscillations in this system. How a completely excitatory network can exhibit the oscillations observed here, including the regular bursting and inter-burst intervals, will be an interesting subject for further functional analysis. A number of labs have now generated MGE-derived cortical interneurons *in vitro* (Liu et al., 2013; Maroof et al., 2013; Nicholas et al., 2013). Mixed cultures of cortical excitatory and inhibitory neurons should allow us to investigate the role of interneurons in the development of cortical circuits *in vitro*, as well as any role in network oscillations.

Stem cell-derived cortical neurons express a range of neurotransmitter receptors, consistent with the receptors expressed by cortical neurons *in vivo.* These include AMPA and NMDA receptors, GABA-A and -B receptors and receptors for a variety of neurotransmitters, including dopamine and acetylcholine. Those receptors are functional in these neurons: AMPA- and NDMA-receptor antagonists inhibit a large fraction of the neuronal activity in stem cell-derived networks, as do GABA-A and GABA-B agonists. Furthermore, stem cell-derived networks support NMDA-receptor mediated plasticity, in the form of chemical LTP. Therefore, we conclude that stem cell-derived neurons form synaptically connected, excitatory networks, the activity of which can be modulated by multiple neurotransmitters, as occurs in the *in vivo* cerebral cortex.

In addition to characterising the functional properties of iPSC-derived human cortical neural networks, we carried out studies of the nature of physical connectivity in this system in order to understand how this would contribute to the observed network properties. Using rabies virus-based trans-synaptic tracing (Wickersham et al., 2007), we found that the majority of cortical neurons in this system receive inputs from fewer than 10 neurons, with a small minority receiving large numbers of inputs. Thus, the overall distribution of connectivity follows a classic power-law. The highly connected group of neurons are typical of hub-like neurons, which would serve to propagate neuronal activity efficiently between areas of the culture, coupling their firing rates.

From our analyses of network formation and function from human pluripotent stem cells, we conclude that these systems have notable utility for fundamental studies of network formation, as well as comparative studies of network properties in health and disease. As we extend these investigations to laminated 3D systems, a key question for these approaches is whether it is possible to exploit developmental biology to construct circuits that reflect those found in the developing human cortex *in vivo*, including canonical cortical microcolumn circuits.

## MATERIALS AND METHODS

### Directed cortical differentiation of hPSCs

Cortical differentiation of human pluripotent stem cells was carried out essentially as described by Shi et al. (2012a,c), with minor modifications. Pluripotent stem cell lines used were CRL, BBHX and JRO (Rashid et al., 2010; Vallier et al., 2009), 2F8 (A. Smith, Cambridge), NDC1.2 (Israel et al., 2012).

### Immunofluorescent staining and microscopy

Cells were fixed in 5% PFA for 10 min at room temperature or in cold methanol at −20°C for 10 min, and blocked for 1 h in 5% donkey serum before overnight incubation in primary antibody. Primary antibodies used: Tbr1 (Abcam, ab31940; 1:500), Tuj1 (Covance, MMS-435P; 1:2000), GFAP (Abcam, ab4674; 1:1000), Otx1/2 (Millipore, AB9566; 1:200), phospho-H3 (Abcam, ab10543; 1:1000), GFP (Abcam, ab13970; 1:1000), TagRFP (Evrogen, AB233; 1:1000). Secondary antibodies conjugated with Alexa Fluor 405, 488, 546, 647 (Life Technologies) were applied after removal of primary antibody and incubated at room temperature for 1 h. Nuclei were stained with DAPI (Sigma). Confocal microscopy was performed on Leica Sp5, Olympus FV1000 Upright or Olympus-19-FV1000 Inverted microscopes. Two-photon microscopy was performed on an upright LaVision TriM Scope II multiphoton microscope equipped with a 20× objective (XLUMPlanFl 20×, NA=0.95; Olympus). Image stacks (505×505 pixels, pixel size 0.792×0.792) were acquired every 1 mm along the *z*-axis. Images were analysed and processed in Volocity (PerkinElmer) and ImageJ (NIH).

### RT-PCR

RNA was extracted using Trizol (Life Technologies) and DNase-treated. cDNA was prepared using SuperscriptII (Life Technologies) and random hexamers. PCR was performed on cDNA using intron-spanning primers (supplementary material Table S2).

### Electrophysiology

Whole-cell current-clamp recordings were performed at 22°C in artificial cerebral spinal fluid bubbled with 95% O_2_ and 5% CO_2_. Borosilicate glass electrodes (resistance 6-10 MΩ) were filled with intracellular solution containing 135 mM potassium gluconate, 7 mM NaCl, 10 mM HEPES, 2mM Na_2_ATP, 0.3 mM Na_2_GTP and 2 mM MgCl_2_. pH adjusted to 7.2-7.4 with KOH. Cells were viewed using a BW50WI microscope (Olympus) or an Axioexaminer A1 (Zeiss) with infrared differential interference contrast optics. Membrane potential and input resistance were recorded after formation of a whole-cell patch. To detect action potential firing, stepwise current injections from −10 pA up to +60 pA in steps of 5-10 pA were performed in current clamp mode. Recordings were made with a Multiclamp 700 A amplifier (Molecular Devices). Signals were filtered at 6 kHz, sampled at 20 kHz with 16-bit resolution, and analysed using custom software in Matlab (MathWorks).

### Calcium imaging

Calcium imaging was performed on CRL and NDC1.2 iPSC-derived cultures aged between 7 and 22 weeks. Six independent wells from each cortical induction were recorded from every 7 to 10 days for analysis by calcium imaging. The same wells were analysed periodically over a ∼100 day timeframe. Cells were loaded with the calcium indicator Oregon Green 488 BAPTA (OGB) by diluting a 0.7 mM OGB stock solution to 3.2 µM in N2B27 media containing 0.01% v/v Cremaphor EL and 0.4% Pluronic F-127 to create a loading solution. Cells were then bathed in the OGB loading solution for 1 h at 37°C and 7% CO_2_ in the dark. The loading solution was then removed and the cells were washed over twice with N2B27 and incubated for a further 30 min at 37°C and 7% CO_2_ in the dark in N2B27. The cells were then imaged in aCSF using a Deltavision (Applied Precision), with an EMCCD camera and using softWoRx 5.0.0 (Applied Precision) software. The imaging chamber was heated to 37°C and supplied with 5% CO_2_. Three independent areas were sampled per well, with three movies recorded per area. Recordings were 2 min in length and were captured at 10 Hz. For pharmacological experiments, cells were perfused with ASCF containing working concentrations of the following drugs/toxins: R-baclofen 50 µM (Tocris Bioscience), bicuculline 20 µM (Sigma-Aldrich), CNQX 50 µM (Tocris Bioscience), DL-AP5 (APV) 50 µM (Tocris Bioscience), muscimol 50 µM (Tocris Bioscience) and tetrodotoxin 1 µM (Tocris Bioscience). The movies were converted to AVI format, accelerated to play at 150 frames per second using ImageJ software and analysed using custom Matlab code (Mathworks, Image Processing Toolbox).

### Lentivirus production

Third-generation replication-incompetent lentivirus was produced by calcium phosphate transfection of HEK293T cells with the lentivirus expression plasmids pBOB-GFP, pBOB-SynP-HTG, complemented with the packaging plasmids pRSV-Rev and pMDLg/pRRE and the VSVG envelope plasmid pMD2.G.

### Analysis of dendritic spines

To analyse dendritic spines, cells were infected with low-titre GFP-expressing lentivirus at day 35. Cultures were fixed on day 40 and 110, and imaged using confocal microscopy.

### Monosynaptic trans-synaptic tracing

2F8 hPSC cortical cultures were infected at day 60 with low-titre TVA-GFP-expressing lentivirus and cultured for a further 5 days. On day 65, cells were infected with TagRFP-expressing pseudotyped rabies (Luo et al., 2008; Rancz et al., 2011) (provided by Troy Margrie, NIMR, Mill Hill, UK) and cultured for 5 more days. Cultures were fixed on day 70, and immunocytochemical staining was performed for GFP and TagRFP and imaged using confocal microscopy.

### Data analysis

To detect recurrent patterns within complex activity, we analysed the order of initiation of calcium transients within large collections (50-100) of cells. Each cell was labelled with a unique ID number, and the temporally ordered sequence of IDs of activated cells was compiled. This time series was then embedded, i.e. a matrix was computed, the rows of which represent the ordered sequence of all subsequences of length *m* (the embedding dimension, usually between 3 and 10). The distance between any one subsequence and another is simply the number of non-identical corresponding members: two fully identical subsequences thus have a mutual distance of zero. The recurrence plot displays a matrix where the distance between subsequence *i* and subsequence *j* is plotted at position (*i*,*j*), as a pixel the colour of which encodes the distance. For recurrence plot quantification, the distance is thresholded, and element (i,j) of the recurrence matrix is set to 1 if the distance between subsequences *i* and *j* is less than the threshold, otherwise to zero. Deterministic dynamics leads to close similarity of trajectories over several successive time steps, i.e. if subsequence *i* is similar to subsequence *j*, then it is likely that subsequence *i+1* will also be similar to subsequence *j+1*, and this is reflected in diagonal lines of slope 1 of high similarity in the recurrence plot (see Fig. 5 for examples). The total recurrence can be quantified by summing the recurrence matrix, and the determinism can be quantified by summing the values which lie within diagonals of length two or greater. The significance of these measures can then be computed by comparison with many randomly shuffled instances (surrogates) of the time series. See Marwan et al. (2007) for details of the method.

The frequency distribution of the number of presynaptic connections made by neurons was fitted under three different assumptions. First, if cells connect randomly with low probability to others drawn from a large population, then the probability of a cell having k connections would be expected to follow a Poisson distribution: 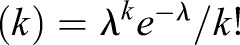, where the single parameter λ is the mean number of connections per neuron. Second, if connections were formed randomly, but from a small set of *n* neurons (e.g. near neighbours) with probability *P*, then 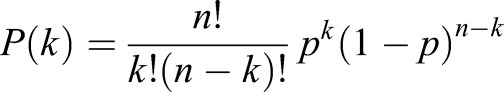, with two parameters, *n* and *P*, forming a binomial distribution. Finally, if the distribution is scale-free, it follows a power law of the form 
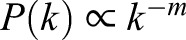
, with a characteristic exponent *m*. These three different models were fitted by least squares to the data.

## Supplementary Material

Supplementary information
