# Supplementary material for: Development and function of human cerebral cortex neural networks from pluripotent stem cells in vitro
Source: Development. 2015 Sep 15;142(18):3178–87. doi: 10.1242/dev.123851 (PMC4582178; doi:10.1242/dev.123851)
Supplement: Supplementary information [file supp_142_18_3178__index.html]

Supplementary information 

# Development and function of human cerebral cortex neural networks from pluripotent stem cells *in vitro*

## DEV123851 Supplementary information

- Supplementary information
