# Supplementary material for: Development and function of human cerebral cortex neural networks from pluripotent stem cells in vitro
Source: Development. 2015 Sep 15;142(18):3178–87. doi: 10.1242/dev.123851 (PMC4582178; doi:10.1242/dev.123851)
Supplement: Supplementary information [file supp_142.18.3178_DEV123851supp.pdf]

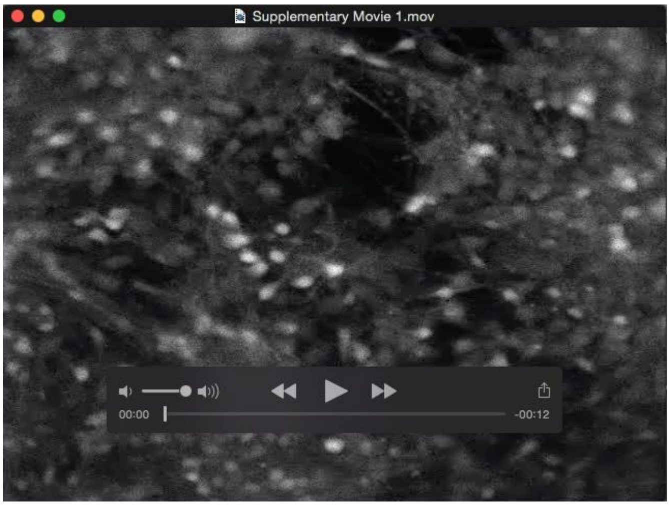

Supplementary Movie 1: Calcium imaging recording of day 58 cortical neurons showing a single synchronised burst.

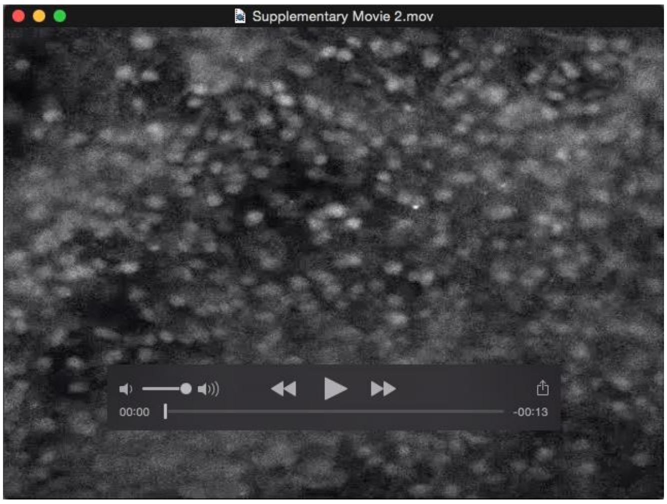

Supplementary Movie 2: Calcium imaging recording of day 64 cortical neurons showing a multiple synchronised bursts.

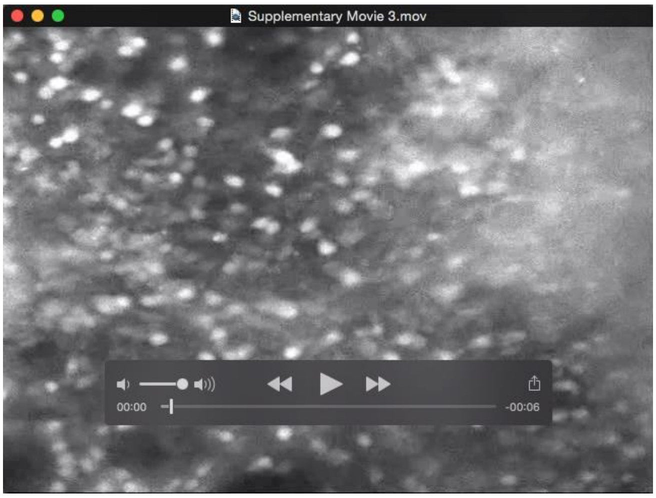

Supplementary Movie 3: Calcium imaging recording of day 70 cortical neurons showing sustained synchronised bursts.

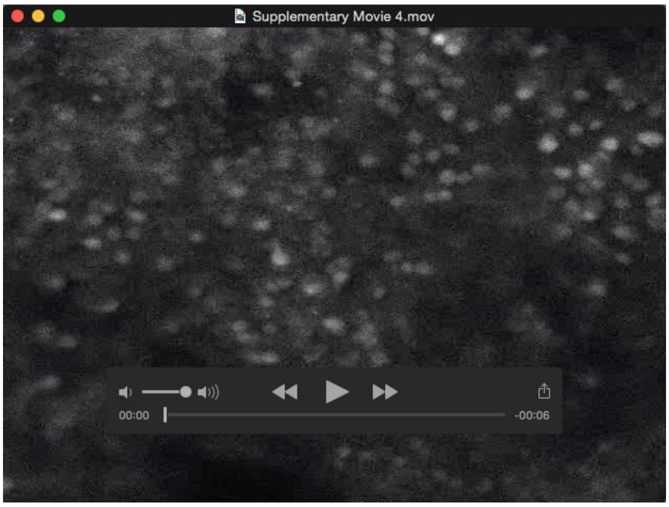

Supplementary Movie 4: Calcium imaging recording of day 77 cortical neurons showing multiple synchronised bursts.

Development • Supplementary information

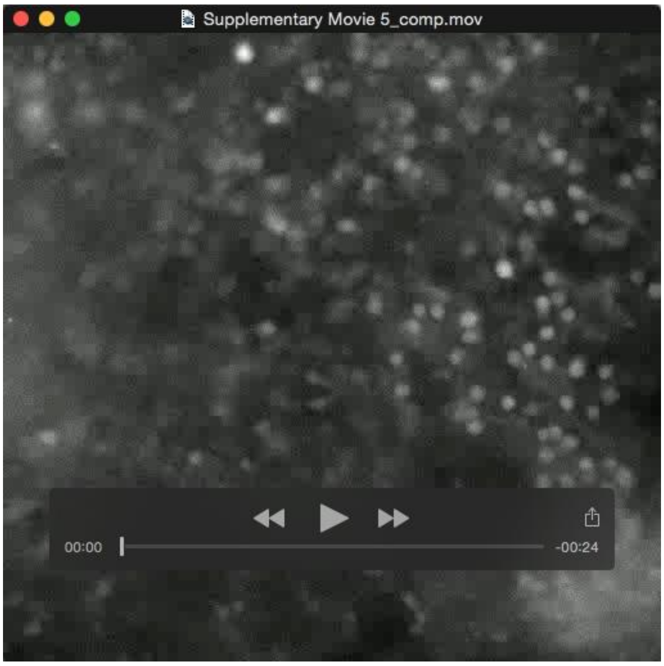

Supplementary Movie 5: Calcium imaging recording of day 108 cortical neurons showing complex network activity.

Supplementary Table 1: Breakdown of whole cell electrophysiological properties.

**BBHX**				
**Day**	**63**	**69**	**76**	**78**
**Resting Membrane Potential**	-30	-50	-42	-45
**mV**	-44	-28	-38	-45
	-45	-38	-43	-54
	-35	-38	-54	-40
	-48	-41	-43	-43
	-37		-50	-40
**Action Potential Height**	10	58.9	56	50
**mV**	31.05	50.2	82	49.9
	54.9	44.5	63	45
	43	56	64	51.1
	25.2	55.4	66	51
	31.1	55.6	73	48
**Number of spikes**	1	5	1	2
**(1 second 10pA stimulation)**	1	3	3	3
	1	1	2	1
	1	1	3	1
	1	1	7	6
	1	2	3	3
**Input resistance**	7.5	3.4	4.5	0.8
**gΩ**	3	4.5	2.6	3.9
	4.1	4.2	1.4	3.6
	1.8	3.2	3.5	2.4
	3.3	4.1	5.5	3.5
	2.1	3.9	2	3.3
**CRL**				
**Day**	**50**	**73**	**100**	
**Resting Membrane Potential**	-35	-43	-52	
**mV**	-48	-53	-50	

Development • Supplementary information

	-50	-47	-58	
	-38	-52		
	-55	-47		
	-40	-57		
**Action Potential Height**	60	49.6	64.6	
**mV**	40.6	57.1	62.9	
	60.3	56	66.4	
		51.8		
		58.1		
		50.1		
**Number of spikes**	1	1	2	
**(1 second 10pA stimulation)**	2	2	3	
	1	2	13	
	1	2		
	1	4		
		3		
**Input resistance**	7.5	4.6	3.3	
**gΩ**	5.6	2.8	3.9	
	6.1	4	2.3	
	6.2	4.1		
		3.5		
		3.8		
**2F8**				
**Day**	**85**	**96**		
**Resting Membrane Potential**	-63	-51		
**mV**	-46	-56		
	-50	-53		
	-35			
	-42			
**Action Potential Height**	70.4	69.4		

Development • Supplementary information

**mV**	61.2	69		
	45	70		
	39			
	75.9			
**Number of spikes**	2	5		
**(1 second 10pA stimulation)**	4	6		
	3	6		
	1			
	3			
**Input resistance**	3.6	3.4		
**gΩ**	3	1.5		
	2.8	2.4		
	3.5			
	3.1			
**JRO**				
**Day**	**71**			
**Resting Membrane Potential**	-50			
**mV**	-60			
	-63			
	-62			
	-30			
	-42			
**Action Potential Height**	66			
**mV**	44.9			
	61.7			
	67.3			
	49.2			
	50			
**Number of spikes**	4			
**(1 second 10pA**	1			

Development • Supplementary information

**stimulation)**				
	3			
	2			
	1			
	1			
**Input Resistance**	3.8			
**gΩ**	2.5			
	5.3			
	3.4			
	3.1			
	5.2			

Development • Supplementary information
